# Supplementary material for: The economic costs of planting, preserving, and managing the world’s forests to mitigate climate change
Source: Nat Commun. 2020 Dec 1;11:5946. doi: 10.1038/s41467-020-19578-z (PMC7708837; doi:10.1038/s41467-020-19578-z)
Supplement: Supplementary file 1 — Supplementary Information [file 41467_2020_19578_MOESM1_ESM.pdf]

Supplementary Information for "The economic costs of planting, preserving, and managing the world's forests to mitigate climate change"

Austin et al.

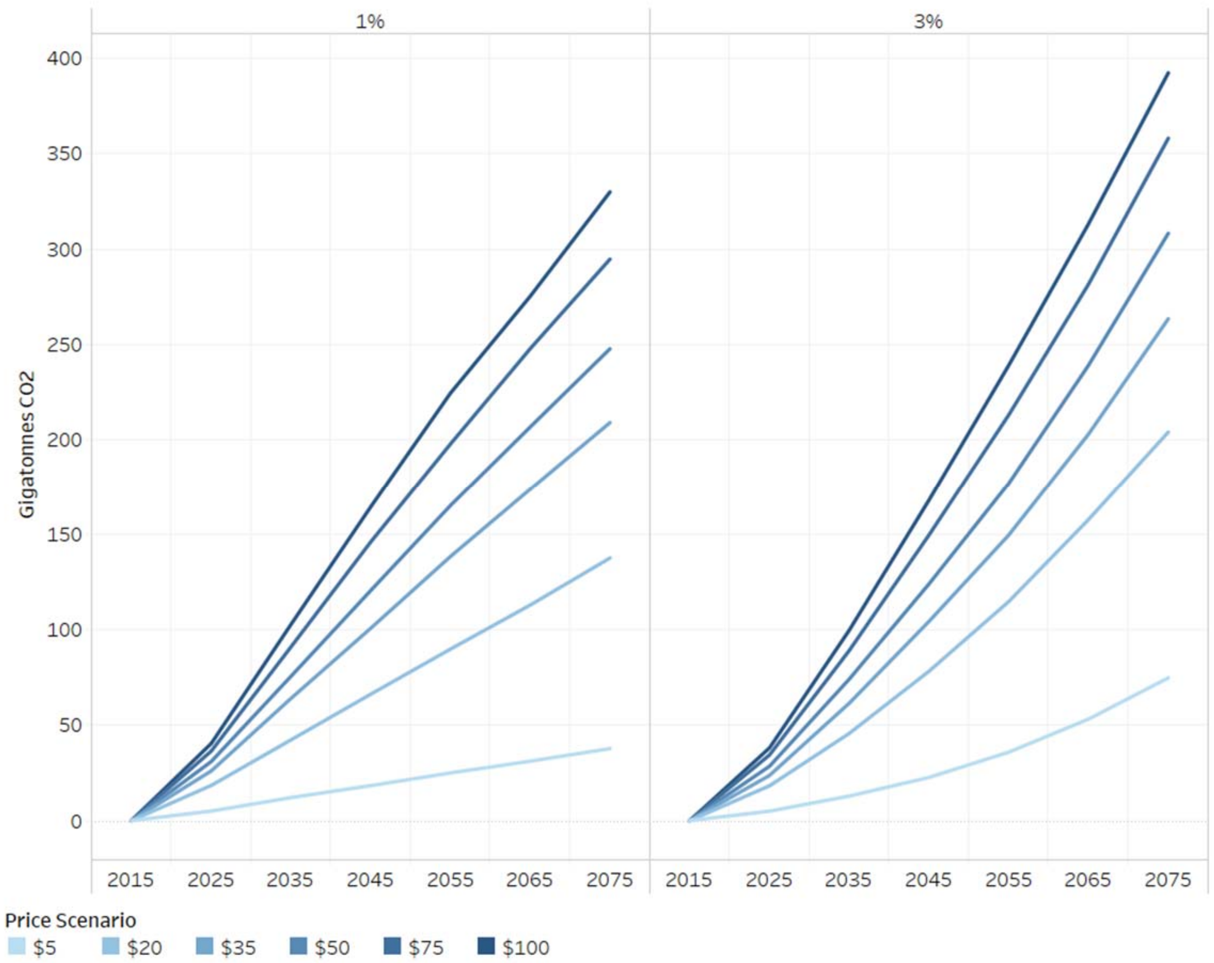

Supplementary Figure 1. Cumulative GHG mitigation across all modelled abatement activities in the global forest sector 2015 - 2075, across carbon price and growth scenarios (all mitigation levels are projected relative to the same baseline simulation with no mitigation incentive).

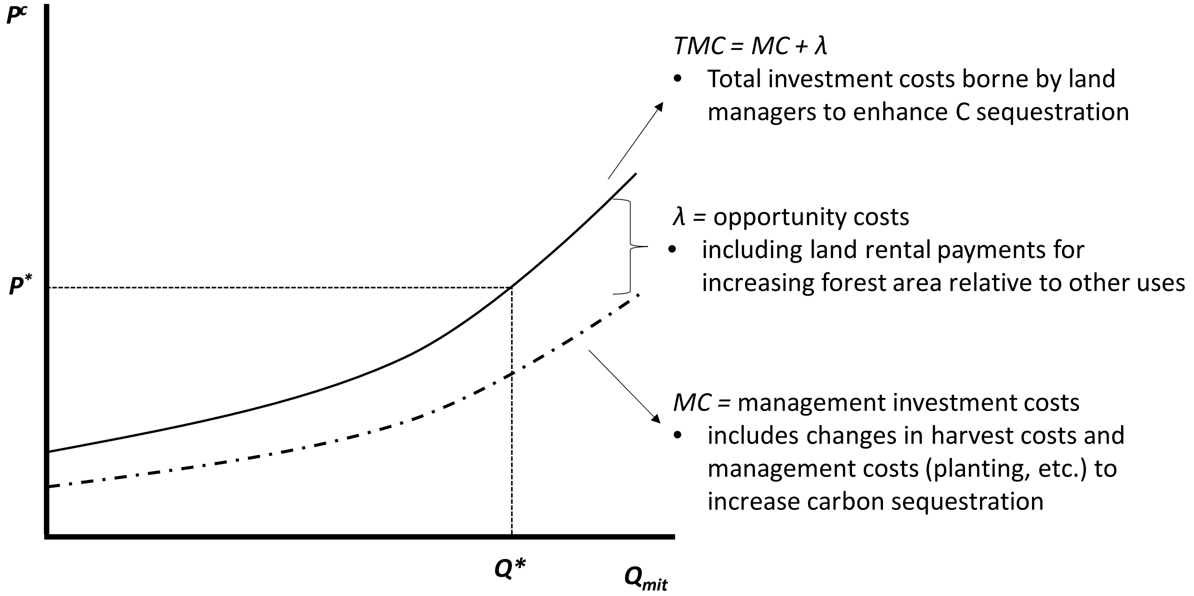

Supplementary Figure 2. Conceptual diagram depicting different sources of costs borne by land managers and comparison to total mitigation costs for a specific price and quantity threshold ($P^* \cdot Q^*$)
