# Supplementary material for: The economic costs of planting, preserving, and managing the world’s forests to mitigate climate change
Source: Nat Commun. 2020 Dec 1;11:5946. doi: 10.1038/s41467-020-19578-z (PMC7708837; doi:10.1038/s41467-020-19578-z)
Supplement: Supplementary file 3 — Description of Additional Supplementary Files [file 41467_2020_19578_MOESM3_ESM.pdf]

Description of Additional Supplementary Files

File Name: Supplementary Data 1
Description: Projected average annual greenhouse gas mitigation in 2035 and 2055 by GTM region under carbon price and growth rate scenarios, in aggregate (A) and by mitigation activity including afforestation (B), avoided deforestation (C), forest management (D) and harvest rotation lengthening (E). Aggregate projected investment required to achieved mitigation quantities in 2035 and 2055 by GTM region and under carbon price and growth rate scenarios (F).

File Name: Supplementary Data 2
Description: Projected average annual harvest of sawlogs and pulplogs by GTM region in the baseline and under carbon price and growth rate scenarios.

File Name: Supplementary Data 3
Description: Projected change in forest and tree plantation area by GTM region in the baseline and under carbon price and growth rate scenarios.

File Name: Supplementary Data 4
Description: Comparison of sawlog and pulplog harvests between average FAO reported values over 2005 – 2015 and GTM reported values in 2015.
